# Management of ecosystems alters vector dynamics and haemosporidian infections

**DOI:** 10.1038/s41598-019-45068-4

**Published:** 2019-06-19

**Authors:** Willem van Hoesel, Alfonso Marzal, Sergio Magallanes, Diego Santiago-Alarcon, Sergio Ibáñez-Bernal, Swen C. Renner

**Affiliations:** 1 Institute of Zoology, University of Natural Resources and Life Sciences, Gregor-Mendel-Straße 33, 1880 Vienna, Austria; 2 Department of Zoology, University of Extremadura, Avenida de Elvas s/n, 06006 Badajoz, Spain; 3 Red de Biología y Conservación de Vertebrados, Instituto de Ecología, A.C. Xalapa, Veracruz, Mexico; 4 Red de Ambiente y Sustentabilidad, Instituto de Ecología, A.C. Xalapa, Veracruz, Mexico; 5 Smithsonian Conservation Biology Center, National Zoological Park, 1500 Remount Road, Front Royal, VA 22630 USA

**Keywords:** Biodiversity, Risk factors, Ecological epidemiology, Forest ecology, Ecosystem ecology

## Abstract

The presence of insect vectors is a key prerequisite for transmission of vector-borne disease such as avian haemosporidians. In general, the effects of land use change on Diptera vectors are not well studied; the response of vectors to forest management depends on vector species, as has been shown previously for the birds. We tested if abundance of insects from different Diptera families and haemosporidian infection are affected through alteration of habitat structural variables (measured by LiDAR) and forest management intensities. We identified higher large-scale variation of female insect abundance in northeastern than in southwestern Germany. Unmanaged forest stands had higher Diptera insect abundances. We found that abundance of female Diptera increased with the amount of forest gaps but decreased in forest plots with more south facing aspect, higher habitat structural heterogeneity, temperature and humidity. We found that haemosporidian infections in Diptera insects increased with increased management intensity and more canopy structural diversity (e.g., amount of edge habitat), but decreased with a denser shrub layer, deeper leaf litter and higher humidity (characteristics for unmanaged forest stands). Although higher forest management intensity decreased vector abundance, the haemosporidian infections in the vectors increased, indicating a significant effect of forest management on disease dynamics.

## Introduction

Habitat alteration and land use intensification are probably the most important drivers for biodiversity loss^1^. Modification of ecosystem processes through, for instance, increased intensity in forest management regimes can affect human and wildlife health^2,3^. Increased intensity in forest management can lead to a higher exposure to diseases through increased transmission risk via changes in resource availability for vectors, hosts and parasites^3–7^. However, it is poorly known how the abundance and composition of dipteran vectors respond to forest structure, in particular to forest use and management regimes^8–12^. Forest management regimes should be a driver for the prevalence of vector-borne diseases because their direct influence on hosts health (e.g., via resource quality and availability) can change their response to parasitism^13^, or because it may alter the availability of suitable habitat for the insect vectors in which the host live^7^. In general, forest structural heterogeneity leads to local variation of vector abundance and species composition as a result of variation in the presence of optimal reproductive habitats^6,14,15^. Spatial and temporal variations in the abundance and the species composition of the vectors results into spatial variation in blood parasite infections and prevalence in vertebrate host populations^16^.

Haemosporidians are vector-borne parasites infecting a broad range of vertebrates worldwide; haemosporidian infections typically result in detrimental consequences for hosts^17^. The order Haemosporida includes malaria-like parasites of the genera *Haemoproteus*, *Leucocytozoon* and *Plasmodium*, of which the latter causes human malaria^17^. These parasites need vertebrate hosts for the asexual stages of their development, while the sexual stages occur within the insect vectors^17^. There are both general and specific relationships between parasite species, vectors and avian hosts^18^, but diversity patterns of haemosporidians resemble that of avian hosts^19^. Each of the vector families transmitting haemosporidians has specific requirements of aquatic habitats for oviposition and larval development^17,20,21^. While larvae of the Simuliidae (black flies) require clean running water, those of Culicidae (mosquitoes) and Ceratopogonidae (biting midges) cover habitats from stagnant aquatic to humid semi-terrestrial habitats, including succulent plants in arid environments^20^. Semi-terrestrial habitats include leaf litter where the larvae feed on, for example, algae or other larvae^22^. Leaf litter is an important reproductive habitat for some vector species^23^ (e.g., culicid vectors) and is directly influenced by tree species composition, which in turn is a consequence of the forest management regimes^24–26^. In addition, canopy trees affect micro-climatic conditions (e.g., temperature, humidity) in the understory of the forest^27^ affecting insect development^28^. Forest management influences microhabitats at fine-scales, for instance by increasing abundance of tree cavities in unmanaged beech forests^29^, which are reproductive habitats for specific vector (e.g. Culicidae^28^) and avian host species (e.g., woodpeckers). Understanding the spatiotemporal variation effects of forest management and structural variables on insect vectors is essential to assess how habitat alterations impact host-vector-parasite dynamics^11,30^ and consequently how parasites can affect wildlife and also humans^11^.

During the second half of the 20^th^ century, European forests have largely changed through increased timber and harvesting intensity^31^. A third of Europe’s land surface is covered by forest^32^, and a quarter of the natural range of European beech forests (*Fagus sylvatica*) is located within Germany covering 4.8% of the German land mass^33^. Additionally, European beech is one of the major tree species in the temperate deciduous forests of Europe^10^. During the 19^th^ and 20^th^ centuries, the main forestry method in beech forests was a shelter wood system. This system extracted mainly large trees, but left chance for overstory trees to regenerate in the clear-cut areas. From the mid-20^th^ century onwards, forestry has focused on more natural-like forest management regimes with the extraction of single trees^34^. As a consequence, forest structure in beech forests diversified and consists of forest stands with multiple layers and age classes^10^. However, whether forest management may alter the transmission risk of avian haemosporidians in insect vector assemblages remains poorly studied.

Here, we study whether forest management regimes and forest structure in beech forests may affect vector abundance and haemosporidian infections in vectors. Specifically, we tested *(i)* how forest management and/or forest structure affects vector abundance at the local forest habitat level, *(ii)* whether vector family/genera species and abundance vary across sites and different years, and *(iii)* how forest management and/or forest structure influence haemosporidian infections in Diptera.

## Materials and Methods

### Study area

Our study is part of the DFG ‘Biodiversity Exploratories’ (www.biodiversity-exploratories.de), a long-term functional biodiversity research program in Germany. The main objective is to study the association between land use intensity and biodiversity, and the expected change in ecosystem processes^35^. In this study, we sampled in two regions, namely “Schorfheide-Chorin” (52°58′N, 13°45′E) and “Schwäbische Alb” (48°24′N, 9°29′E) Biosphere Reserves, henceforth called ‘Northeastern’ and ‘Southwestern’ region, respectively (coordinates of each sampling site are given in Supplementary Information Table S1).

### Forest categories, forest structure, and environmental conditions

In 2014 and 2015, we sampled vectors on 21 forest plots at each region. Forest plots are dominated by European beech *Fagus sylvatica*, representing at least 70% of the canopy inside each plot and can be mixed with other broadleaved species (e.g., *Quercus spp*.) or conifers, mainly *Pinus sylvestris* in the Northeastern region and *Picea abies* in the Southwestern region. We sampled seven separate plots within each of the following three *a priori* determined forest categories: (a) unmanaged beech stands (unmanaged for >60 years), (b) age class young and (c) age class old. Dividing managed forest stands into the two age classes was based on the median age of the respective region (Northeast: 138 years; Southwest: 90 years), e.g., age class young was assigned when the stand age was lower than the median age^35,36^. Each plot covers an area of 100 × 100 meters and is surrounded by the same forest type by a buffer fringe of at least 30 m^35^.

Fine-scale forest structure is frequently determined through Light Detection and Ranging (LiDAR) methods, a remote sensing technique that uses near-infrared to detect structural variation of surfaces, such as vegetation^37^. This technique shows potential to explain insects’ responses to canopy structure^38^ and is increasingly used to assess biodiversity in relation to habitat heterogeneity in forests^39,40^ or microhabitats for vectors^41^. Transmission risk is influenced by both vegetation and vector ecology, and remote sensing techniques offer a range of opportunities to study these dynamics^42^.

We collected LiDAR-derived forest characteristics in the Northeastern region in 2009 and in the Southwestern region in 2010. We selected a subset of LiDAR variables *a priori*, based on their likely ecological relevance to study host-vector-parasite interactions^43^: understory (proportion of understory vegetation cover, up to two meter above the ground), shrub layer (proportion of shrub vegetation cover, between three and seven meter above the ground), south facing (proportion of canopy on south facing slopes), gap (proportion of canopy characterized as gap), edge (proportion of canopy that differs at least five meters in height with the surroundings), open stem zone (from soil surface to the lower part of the canopy) and entropy (a measure for local vertical variation in the canopy, i.e. canopy structure; details on LiDAR variables in Supplementary Information Table S2). In order to disentangle the potentially subtle effects and differences of forest management we used a silvicultural management index indicator (SMI: high values indicate high management intensity) in addition to the coarse forest categories. The SMI takes into account the effects of tree species selection, stand age, biomass removal and the regeneration method^44^. This SMI was specifically designed for the forest stands sampled in our study and is suggested to help assess the impact of management on forest biodiversity^44^.

On every plot, we continuously collected temperature and relative humidity at 200 cm above ground. We used the daily means averaged for the two preceding weeks of the capture day to approximately cover the development period of vectors larval stages^20^. As a measure for breeding habitat of some vector species^23^, we used the depth of the leaf litter layer by randomly selecting ten locations within each plot, both in 2014 and 2015. We probed for leaf litter depth four times at each of those locations and took the average. Additionally, we analyzed reproductive habitat in terms of proximity and size of standing and flowing water, which we extracted from the Digital Landscape Models (DLM) for each region.

### Vector trapping, sorting, and genetic analysis

As part of an ongoing study on the interaction between land use and host-vector-parasite interactions in avian hosts^7,13^, we sampled vectors on the 21 plots, seven plots within each of the three forest categories, during birds’ breeding seasons of 2014 and 2015. In 2014, the sites were sampled from April until the beginning of July (Northeastern site: April 21–June 21; Southwestern site: May 4–July 9). In 2015 the sampling was repeated between May and July (Northeastern site: May 22–June 26; Southwestern site: May 6–July 2). Differences in start and ending were due to alternating weather conditions for insect trapping at the different regions.

For insect trapping we used BG Sentinel Traps placed on the forest floor, baited with CO_2_ (outflow of 200 g per 24 h), Biogents’ Sweet-scent and an ultraviolet module (Biogents AG, Regensburg, Germany). All traps ran for 18 hours, starting typically at 2 pm, until the next morning. We placed the insects in a freezer at −20 °C for at least one hour and transferred the whole capture into 80% analytical grade ethanol. We sampled once on each of the 21 selected plots in each region per year.

The collected insects were sorted using a key for European Diptera to separate the target families: Culicidae, Simuliidae, and Ceratopogonidae^22^. We separated all individuals to groups based on their morphology (i.e., sorted to morphospecies), per plot and per year. For species identification and parasite detection (through PCR and sequencing), we took one female individual per group. From the groups containing Ceratopogonidae, we only selected female individuals of the genus *Culicoides*, which are known to transmit haemosporidians to avian hosts^17,21^ and are typically characterized by their spotted wings^45,46^. The selection of one individual per group resulted in 220 analyzed individual specimens for species identification and the screening of an infection with avian haemosporidians. The detected parasite infections among the tested samples therefore represent a relatively low-resolution of the actual parasite presence within the vector community.

From each individual specimen, we dissected head and thorax to exclude the abdomen for analysis of infections because a positive detection from the mid-gut might represent parasite DNA from a recent blood meal and does not indicate infectiveness of a vector towards a bird host^20,47^. After dissection, we crushed the combined head and thorax using a pellet pestle and added a total of 180 µl of digestion solution (Thermo Scientific™ K0722) and we extracted DNA using the protocol for isolating genomic DNA (Thermo Scientific™ Genomic DNA Purification Kit K0512). For vector species identification we used the primer pairs LCO1490 and HCO2198 amplifying a ~690-bp fragment of the CO1 gene^48^ in combination with a modified PCR thermal cycle^49^. These primers were also used in other studies for vector species identification^50,51^. For the detection of parasites inside the insect vector, we followed a nested PCR protocol amplifying a ~480-bp fragment of the mtDNA cytochrome-b gene for the detection of the three genera of avian haemosporidian parasites (details in Supplementary Information S1).

For vector species identification, we further used PCR products for sequencing and compared the sequences with the nucleotide-nucleotide basic alignment tool database (BLAST; GenBank DNA sequence database, National Center for Biotechnology Information). We then crosschecked with six separate databases for species’ distribution to see whether identification returned a probable species, foremost with the Walter Reed Biosystematics Unit on Culicidae (www.mosquitocatalog.org) and the work by Knight^52^. Furthermore, we used the BOLD public data portal on findings of the same species^53^, distribution maps on the Interactive Identification Key for *Culicoides*^54^, the vector list of the database of avian haemosporidian parasites MalAvi^55^. For the analysis we excluded entries for which we could not obtain a sequence or if the known distribution of the identified species did explicitly not cover our region (Germany) to increase confidence of the identification. From the originally 220 analyzed individual specimens, we used 189 for further analysis. All dipteran species identified in this study are known to take blood meals from warm-blooded vertebrates, often with a special affinity for avian hosts (ornithophilic) and were thus regarded as potential vectors of avian haemosporidians and were included in further analyses (details on the host-affinity of the vector species in Supplementary Information Table S3).

We took samples in accordance with national German and European Union law on animal protection and conservation measures. All needed permits have been approved by the authorities. Permit numbers and the respective authorities are given in the Acknowledgements.

### Statistical analysis

We used Generalized Linear Models (GLM) for the count data of vector samples. We adjusted to negative binomial distribution to correct for the overdispersion initially observed when assuming Poisson distribution^56^. We used a model selection approach by using the “drop1” command^57^, which compares the deviances of models by dropping each predictor variable. We stepwise removed one predictor variable with the highest *P*-value until we got a nested model with predictor variables with *P* < 0.05^56^. We then selected the best-fitted model based on the largest difference of the Akaike Information Criterion corrected for small sample sizes (ΔAICc) to the full model, regarding a better model-fit with decreasing AICc from the initial model^58^. We used the “model.sel” function from the “MuMIn” package to acquire model estimates^59^. We used the “effects” package to visualize the effects of significant predictor variables^60^. All data were analyzed using R version 3.3.3^57^.

The full model for female vector abundance was: vector abundance ~SMI + forest category + understory + shrub layer + south facing + gap + edge + open stem zone + entropy + distance to standing water + distance to flowing water + size of standing water + depth of leaf litter layer + temperature + relative humidity + genus + region + year of capture (predictor variables are detailed in Supplementary Information Table S2). We repeated the same full model but using binomial GLMs with the same predictor variables and parasite infection as the response variable.

For female vector abundance, we analyzed the dataset in two different ways: we first analyzed the dataset for the whole vector community; then, we divided the dataset based on Diptera vector family because they have different reproductive biology and habitat requirements^20^. The second analysis only included data for Ceratopogonidae (biting midges) and Culicidae (mosquitoes), because the sample size for black flies (Simuliidae) was small.

## Results

### Total abundance of female vectors and species numbers

We captured a total of 4,759 female potential vectors and 1,743 male individuals in the three different forest categories (unmanaged: 1,557 females/469 males; age class young: 1690/634; age class old: 1,512/640) in the two years. Genetic analysis resulted in 16 species of Ceratopogonidae (biting midges, only of the genus *Culicoides*), 12 species of Culicidae (mosquitoes) and four species of Simuliidae (black flies; details in Supplementary Information Table S4). For Ceratopogonidae, the most common species were *Culicoides pictipennis, Culicoides festivipennis* and *Culicoides impunctatus*. For Culicidae, the most common species were *Anopheles plumbeus*, *Aedes cantans* and *Aedes punctor*.

From the 189 tested individual specimens, 84 tested positive for avian haemosporidians (44%); 76 females were infected by either *Haemoproteus* or *Plasmodium* lineages, eight of them by *Leucocytozoon* lineages and three carried a mixed infection (details in Supplementary Information Table S5).

### Effects of forest structure on female vector abundance

**Figure 1:**
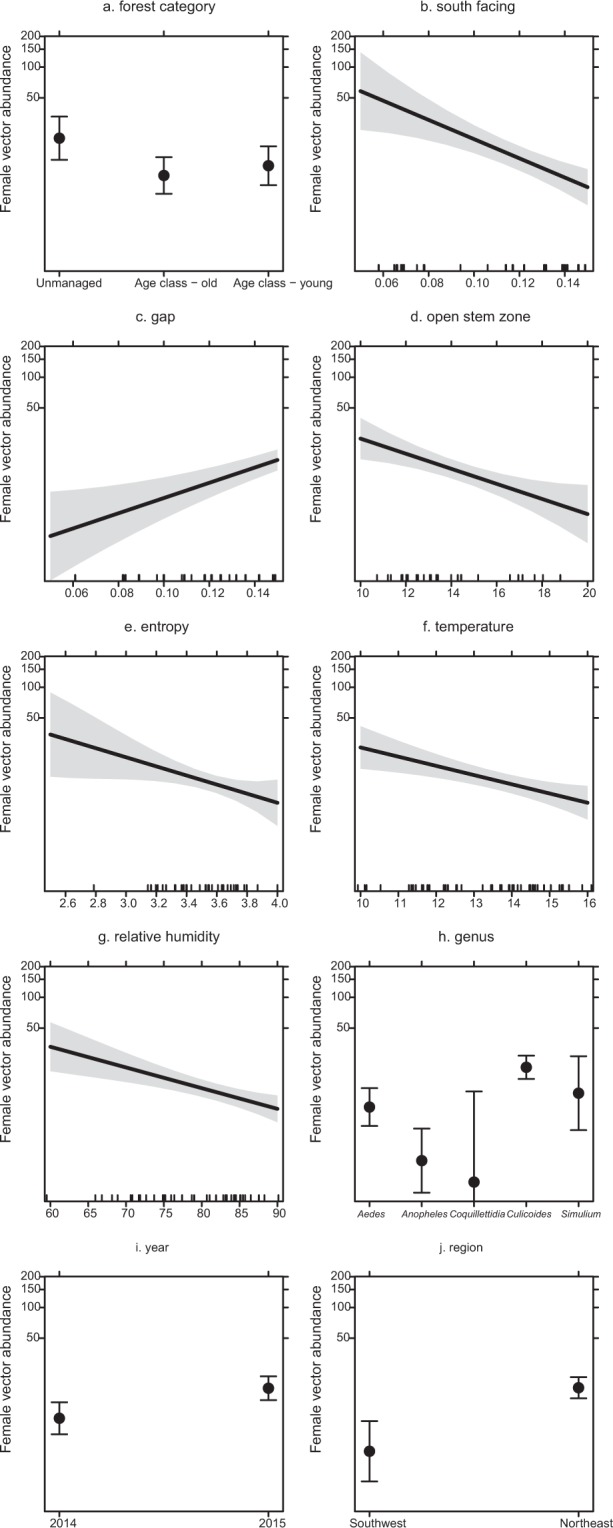
The effect of the significant predictor variables on total vector abundance (±95% CI). Shown are values predicted using the best-fitted model from Table 1. The *y*-axis is *log*-scaled.

The best-fitted model for total female vector abundance (ignoring the factor “vector families”), includes significant negative effects of forest category (being lower in age class old forest stands than in unmanaged stands), south facing, open stem zone, entropy, average temperature, and relative humidity (GLM; *P* < 0.05, Fig. 1, Table 1; all model estimates in Supplementary Information Tables S6–S9). There are significant positive effects on vector abundance by the proportion of gaps (LiDAR: Ga), and significant differences between years of capture and between regions (GLM; *P* < 0.05, Fig. 1, Table 1). We also found differences in the abundance of insect genera, with significantly lower abundances of *Anopheles* mosquitoes compared to the other genera and a significantly higher abundance of biting midges genus *Culicoides* compared to mosquitoes of the genus *Aedes*.

**Table 1:**
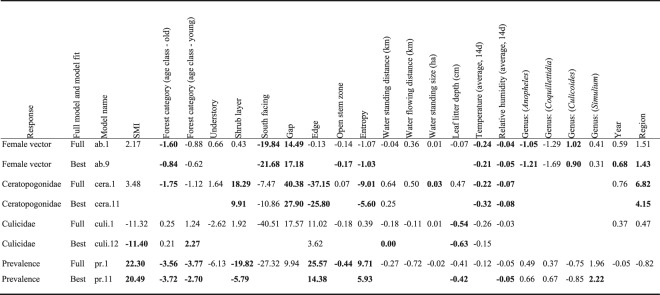
Generalized Linear Model parameter estimates of female vector abundance (whole data set) vector abundance separated per vector family (for Ceratopogonidae and Culicidae), and parasite prevalence in the insect vector for the full and best model. *Blank fields* indicate all parameter excluded in the best-fitting model, further explanations of variables in Supplementary Information Table S2. Estimators in bold indicate significant (*P* < 0.05) estimators.

Based on the large differences in abundances among Diptera families, we separated the analysis per genus: (a) Ceratopogonidae (biting midges) abundance was negatively associated with the proportion of habitat edge, entropy, average temperature and average relative humidity. Ceratopogonidae abundance was positively associated with the proportion of shrub layer and the proportion of forest gaps. There are also regional differences, where the abundance of ceratopogonids was higher in the northeastern region (all GLM; *P* < 0.05, Fig. S1, Table 1). (b) For Culicidae (mosquitoes) abundance, we found a significant negative effect when increasing silvicultural management (SMI), which contrasts with the significant higher abundance of mosquitoes in age class young stands compared to unmanaged beech stands (Fig. S2, Table 1), as both young and old age forest classes have a significantly higher SMI compared to unmanaged stands (Kruskal-Wallis, χ^2^ = 74.036, df = 37, *P* < 0.01, Fig. S3). Leaf litter depth also had a negative effect on culicid vector abundance (all GLM; *P* < 0.05, Fig. S2, Table 1).

### Haemosporidian infection in female vectors

**Figure 2:**
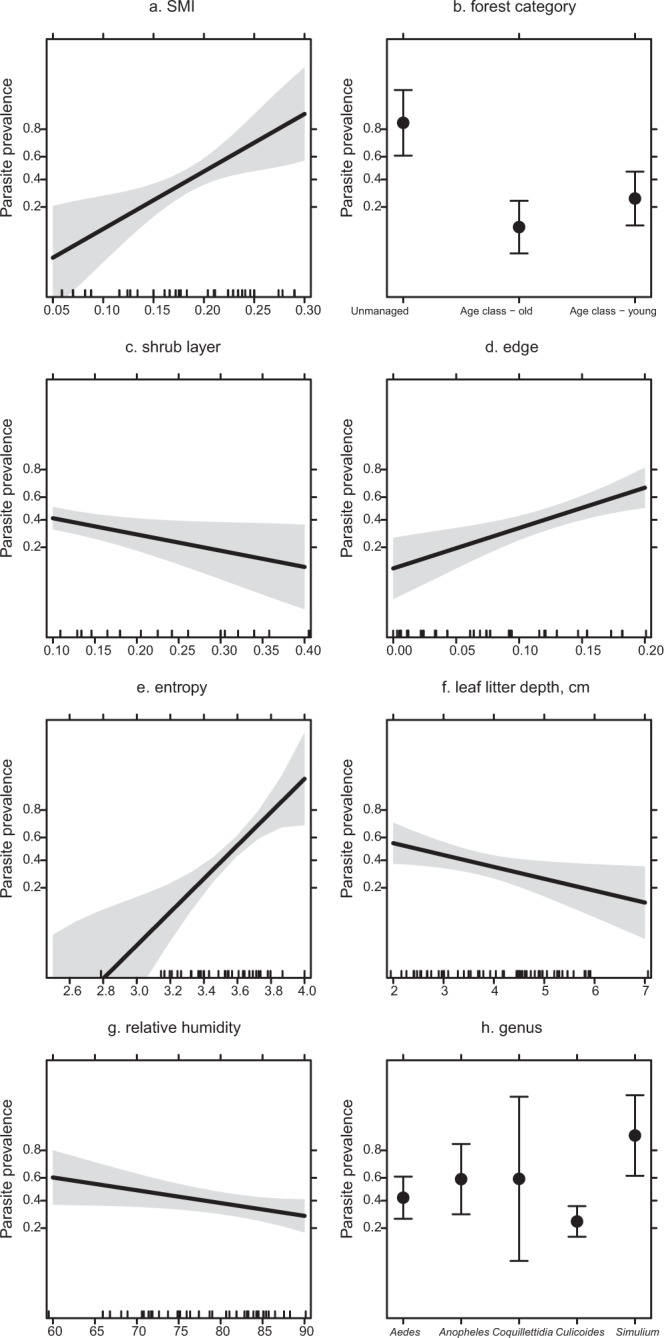
Tshe effect of significant predictor variables on haemosporidian prevalence in insect Diptera vectors (±95% CI). Shown are values predicted using the best-fitted model from Table 1.

For haemosporidian parasite infection of insect vectors, we found negative associations with the proportion of shrub layer, leaf litter depth and relative humidity, whereas we found a significant positive effect of silvicultural management index (SMI) on parasite infection, indicating that stands with higher manipulation harbor more infected individuals. This is in contrast with our finding that vectors in age class forest stands (i.e. managed) had fewer infected individuals compared to unmanaged forest stands. In addition, the proportion of edge and entropy were positively associated with haemosporidian infection in vectors. We found significantly more infections in *Simulium* black flies than in *Aedes* mosquitos and *Culicoides* biting midges (all GLM; *P* < 0.05, Fig. 2. Table 1).

## Discussion

Anthropogenic changes (e.g., urbanization, agriculture, forestry) reduce species richness, change abundance structure, and alter the interaction dynamics of wildlife^1,43^. Recently, information has been generated on how human induced modifications of the environment affect ecological dynamics of vertebrate parasites^7,13^. However, very little is known on how such changes affect the insect vectors involved in the life cycles of different parasite groups^5^. Here, we studied how age forest categories and forest structural variables affected Diptera vectors abundance and haemosporidian infection rates in vectors. We found that old forest stands had significantly lower vector abundance and parasite infections compared to unmanaged forest stands, and that variables, such as south facing slopes, negatively affected the abundance of Diptera insects. Moreover, those forest stands with more heterogeneous habitat structure, a wider altitudinal range with less stems (more open under and over stories), higher average temperature and humidity reduced Diptera abundance. The only variable positively associated with vector abundance was the number of gaps in forest plots, which is likely an area providing good breeding sites for insect development (e.g., humidity, puddles, light, and nutrients). In terms of infection rates in the tested Diptera vectors, the amount of edge habitat and habitat structural heterogeneity (i.e., entropy) were positively associated with parasite infections; however, increased shrub density, leaf litter depth and relative humidity negatively affected the probability of infection for insect vectors. We found contrasting effects of the *a priori* determined forest categories compared to the effect of increasing management index, SMI. Even though the coarse categories and the SMI are related (Supplementary Information Fig. S3a), the categories were initially determined to facilitate field sampling. Moreover, the SMI is based on a set of forest properties that reflect management in more detail (i.e., tree species, stand age, above ground biomass and regeneration method)^44^ and therefore this index is likely to have more ecological relevance than coarse categorizations. Therefore, we conclude that forest stands with higher management intensity (i.e., higher SMI) harbor more infected vectors compared to stands with lower management intensity. Our results on the fine-scale management differences extend previous findings on effects of coarse management modifications (such as deforestation and urbanization) on variation in vector abundance and infection dynamics^11,12,15,61,62^.

Forest structure affected female ceratopogonid vectors, because they were more abundant in forest stands with a rather uniform canopy, but with a higher structural diversity in the shrub layer. Canopy structure affects insect abundance and the abundance of specific Diptera families was found to be positively affected by increased vegetation density in both lower and upper vegetation layers^63^. Similarly, other studies have found a positive relationship between increasing vertical distribution of biomass and the abundance of arthropods, particularly Diptera^39,64^.

We found higher female ceratopogonid vector abundance in the Northeastern compared to the Southwestern region, confirming site differences at broader geographic scales. The variations between the sites are manifested in differing geological history, local climate, and local availability of water^65,66^. The Northeastern region is a young lowland glacial landscape with many aquatic habitats, whereas the Southwestern region consists of calcareous mountain ranges with very few standing water^35^. Although the Northeastern region has a lower annual rainfall than the Southwestern region (500–600 mm vs. 700–1000 mm), the amount of lakes and ponds is larger in the northeast^35^. A higher lake and pond abundance in the northeast may explain the higher abundance of female ceratopogonid vectors of the genus *Culicoides*, whose immature stages develop in a wide range of aquatic habitats, such as lake margins^20^. Although not measured directly in this study, there could be a direct effect of annual rainfall on abundance of ceratopogonid vectors such as *Culicoides imicola*, whose occurrence is restricted to regions with annual rainfall between 300–700 mm, as higher precipitation rates could lead to the drowning of pupa^67–69^. We found a negative effect of increasing temperature on ceratopogonid abundance, contrasting with common perception of a generally positive effect of temperature on development of immature stages^70,71^ or adult vector activity in general^20^. However, very hot/dry and very wet/humid environmental conditions negatively impact *Culicoides* abundances^72^, as survivorship of adult Ceratopogonidae is lower at higher temperatures^73,74^. As most *Culicoides* are crepuscular or nocturnal, adults are mostly active with mild temperatures^74^, which is congruent with our findings. We observed negative effects of relative humidity on female ceratopogonid abundance; because warmer air can hold more moisture, adult vectors risk desiccation^74^. Therefore, a combined higher air temperature and higher relative humidity could represent suboptimal flight conditions for ceratopogonid vectors, reflected in their negative effect on *Culicoides* abundance.

Culicid vectors responded different compared to Ceratopogonidae. We found that age class young forests have higher mosquito abundances, compared to age class old and unmanaged stands. At the same time, we found a negative effect of increasing management intensity index (SMI). The age class forest (i.e., managed) stands in this study have a higher silvicultural management index, than unmanaged forest stands (Supplementary Information Fig. S3a)^44^. While we find that age class young forest stands (which have a higher SMI compared to unmanaged stands) have higher culicid abundance, at the same time we see a negative effect of increasing SMI (Supplementary Information Fig. S2) – which seems to be a contradiction, like with parasite infections. Again, the forest categories we used coarsely reflect forest management; we decided on their age-class *a priori*. In turn, the SMI combines a set of stand characteristics including tree species, stand age, above ground biomass and regeneration method (i.e. planting vs. natural regeneration)^44^, and its effect on culicid abundance better explains the ecological effect of management intensity. For example, higher habitat edge, more open under- and over- stories (i.e., less stems) and a more homogeneous canopy structure imply less above ground biomass, suggesting fewer breeding places for mosquitoes, likely explaining the negative association between SMI and mosquito abundances.

We found a negative effect of leaf litter depth on culicid abundance. Interestingly, larvae of the most abundant mosquito species (*Ae. cantans*, *Ae. annulipes* and *Ae. diantaeus*) occur in water bodies with a significant amount of leaf detritus at the bottom^28^. Larvae of our most common species, *An. plumbeus*, develop mostly in tree holes^28^, but larval development could actually be limited by the quantity of the slowly decomposing beech leaf litter as observed in other mosquito species^75,76^. Thus, the negative effect of increased leaf litter depth on culicid abundance is likely related to the effect that a higher productivity does not *per se* lead to a higher abundance, but rather to a higher species richness^77^. In relation to management intensity and those species that reproduce exclusively in tree holes – both abundance and species richness are negatively affected by increased intensity^78,79^. There was also a negative effect of leaf litter depth on infection rate. Forests with a higher management intensity index (SMI) have typically thinner leaf litter layers and a higher leaf litter turnover rate^80^, which may explain the higher infection rates and lower mosquito abundances on forest stands with higher SMI. Therefore, our results suggest an indirect effect on parasite infections by management intensity (i.e., SMI) via leaf-litter dynamics.

We found a positive effect on mosquito abundance as distance to standing water increased. This is in contrast with other studies showing that particularly Culicidae abundance is best explained by models including proximity to water or the size of standing water^21,66,81,82^. Water bodies locally increase the availability of suitable breeding habitat for vectors^82^ and attract female vectors actively searching for hosts or for suitable oviposition sites^28^. The fact that the studied forests have higher water availability, providing water sources across the forest area, could explain the unexpected association between these variables. In addition, different mosquito species have different breeding requirements, so other habitat variables (e.g., gaps) might be more important for their abundance instead of just water sources.

We found a positive effect of forest structural complexity on parasite infection probability for vectors. We explain our results in two complementary mechanisms: first, heterogeneous canopy structure positively affects the Diptera abundance and species composition via the presence of canopy edges, open areas^83^, and vegetation heterogeneity^9^. Canopy openness and structural variation allow more light to reach the forest interior and undergrowth, generally resulting in warmer microclimates compared to forests with a closed canopy^27,84^. Warmer conditions promote parasite development in both vector and avian hosts^85–87^. Second, infected avian hosts act as parasite reservoirs for the vectors; at the same time structurally diverse areas attract forest birds because of higher resource availability (e.g., increased possibilities on feeding on arthropods)^88–91^. In addition, vectors of avian haemosporidians show increased mating, host-seeking, blood-feeding activities in warmer conditions^28,67^, which in turn can promote the biting rate and infection from an infected avian host.

Combined, a higher detection of parasite infections within the vectors is driven by more diverse canopy structure and an increased chance for encounters between an infected avian host and a vector^92^. For example, parasite prevalence in Eurasian blackcaps, *Sylvia atricapilla*, was positively related to forest stands with higher degree of open areas^7^, which increases the likelihood of infecting the vectors by presence of the infected birds as reservoirs. Although the effects of forest structure on parasite prevalence in hosts are species-specific (e.g., chaffinches, *Fringilla coelebs*, responded different than blackcaps^7,93^ and some vector species have adapted their behavior to finding hosts higher up in the canopy^94,95^), we observed that forest canopy structure positively effects parasite infections in Diptera vectors and negatively their local abundance^96^.

The here presented measure for parasite infections reflects a through estimate of the actual parasite prevalence among the whole vector community, since we tested a single individual and not all. However, the aim of this study was to analyze the effects of land use, i.e. forest management and thus structure, on vector abundance and parasite infections within the vectors. We found that the effects are subtle and are determined through forest structure for both the vector abundance and parasite infections. For future research and increased understanding on vector-parasite-environment interactions, it would be benefical to increase research on vector-parasite associations, particularly potential differences between regions, and regions with varying human influence.

In conclusion, local transmission of avian haemosporidians in insect vectors are driven by several forest stand attributes that also affect Diptera abundance, such effects can vary in direction and magnitude depending on the insect family under study. Ignoring such varying aspects in vector-host-parasite studies may result in low predictability and understanding of ecological dynamics. Assessing suitable habitat for vector reproduction in future studies is important to understand variation of vector abundance, but also the actual presence of infective vectors, whose status is associated with the presence of infected avian hosts as a measure of transmission risk. The critical question in vector-borne disease studies is the understanding of the mediating role of land use regimes on the vectors.

## Supplementary information

Supplementary Information

Raw Data

## Data Availability

The original data is available in Table S10 in the Online Supplementary Information.
