# Supplementary material for: Management of ecosystems alters vector dynamics and haemosporidian infections
Source: Sci Rep. 2019 Jun 19;9:8779. doi: 10.1038/s41598-019-45068-4 (PMC6584559; doi:10.1038/s41598-019-45068-4)

Management of ecosystems alters vector dynamics and haemosporidian infections

Willem van Hoesel, Alfonso Marzal, Sergio Magallanes, Diego Santiago-Alarcon, Sergio Ibáñez-Bernal, Swen C. Renner

Description of Supplementary Information:

Supplementary Information S1

Vectors of avian Haemosporidia
Detailed PCR protocol for vector species identification
Detailed PCR protocol for the detection of parasite infection and identification of the parasites (genus level)

Table S1. Coordinates of each sampling site. Coordinates are given in WGS84 projection.

Table S2. Overview of predictor variables used in the analysis, the *grey shaded* area includes the predictor variables that are obtained by LiDAR.

Table S3. Overview of captured vector species grouped per family for the years 2014 and 2015.

Table S4. Number of female individuals caught per species in each region, separated per year.

Table S5. Overview of vector species with positive detection of haemosporidian infection after PCR.

Table S6. Model estimates from the Generalized Linear Models for total vector abundance.

Table S7. Model estimates from the Generalized Linear Models for female ceratopogonid vector abundance.

Table S8. Model estimates from the Generalized Linear Models for female culicid vector abundance.

Table S9. Model estimates from the Generalized Linear Models for prevalence inside the vector.

Table S10. Raw data (provided as separate .xlsx file)

References cited in Supplementary Information

Figure S1. The effect of the significant predictor variables on the abundance of ceratopogonid vectors (±95% CI).

Figure S2. The effect of the significant predictor variables on the abundance of culicid vectors (±95% CI). Shown are values predicted using the best-fitted model from Table 1.

Fig. S3. Relationships between the Silvicultural Management Index (SMI), forest categories and leaf litter depth for the sampled forest plots.

Supplementary Information S1

Vectors of avian Haemosporidia

The life cycle of avian haemosporidian blood parasites takes place partially in the insect and in the bird hosts, respectively the sexual and asexual stages [1,2]. There are four families of Haemosporidia that develop their asexual stages in birds and are transmitted by different dipteran vectors [2]. Biting midges (Ceratopogonidae) and louse flies (Hippoboscidae) spread Haemoprotidae, mosquitoes (Culicidae) spread Plasmodiidae and Garniidae and black flies (Simuliidae) spread Leucocytozoidae [2-4]. In our study, we did not study hippoboscid vectors as they are ectoparasites on birds [5] and would therefore rarely occur in freely placed traps.

Detailed PCR protocol for vector species identification

After DNA extraction, we quantified the amount of DNA using a spectrometer (Thermo Scientific™ Biomate 3S) and subsequently diluted to an optimal concentration of 5 ng/µl of DNA. We disregarded any samples without detected DNA in all further procedures. We used primer pairs LCO1490 and HCO2198 to amplify approximately 600 bp fragments of the CO1 gene [6]. Each cocktail contained 2 µl of DNA template, 0.25 µl enzyme, 1 µl forward primer (LCO), 1 µl reverse primer (HCO), 2 µl of buffer, 0.4 µl of dNTP and 13.35 µl of double distilled H2O. The total volume constituted to 20 µl. The PCR thermal cycle consisted of one cycle of 3 min at 94°C, 40 cycles of 30 s at 94°C, 30 s at 46°C, 60 s at 72°C and a final cycle of 10 min at 72°C [7].

Detailed PCR protocol for the detection of parasite infection and identification of the parasites (genus level)

To detect a parasite infection inside the vector, we followed a nested PCR protocol for the detection of all three genera of avian haemosporidian parasites: Haemoproteus, Plasmodium and Leucocytozoon [8]. For the first PCR we used the primer pairs HaemNF1 and HaemNR3 for the amplification of parasite mitochondrial DNA. Each cocktail contained 2 µl of DNA template, 0.1 µl enzyme, 1 µl forward primer (HaemNF1), 1 µl reverse primer (HaemNR3), 2.5 µl of buffer, 2.5 µl of dNTP and 15.9 µl of double distilled H2O. The total volume constituted to 25 µl. The PCR thermal cycle for the first PCR consisted of one cycle of 2 min at 94°C, 25 cycles of 30 s at 94°C, 30 s at 50°C, 45 s at 72°C and a final cycle of 10 min at 72°C.

For the second PCR we have used the primer pairs HaemF + HaemR2 and HaemLF + HaemLR2 [8-10]. Subsequently, each cocktail contained 2 µl of the product from the first PCR, 0.1 µl enzyme, 1 µl forward primer (HaemF or HaemLF), 1 µl reverse primer (HaemR2 or HaemLR2), 2.5 µl of buffer, 2.5 µl of dNTP and 15.9 µl of double distilled H2O. The PCR thermal cycle for the second PCR consisted of one cycle of 2 min at 94°C, 35 cycles of 30 s at 94°C, 30 s at 50°C, 45 s at 72°C and a final cycle of 10 min at 72°C.

We followed a nested PCR protocol amplifying ~ 480-bp fragment of the mtDNA cytochrome b gene for the detection of the three genera of avian haemosporidian parasites. For the first PCR we used the primers NF1 and NR3. For the second PCR we used HaemF and HeamR2 for *Haemoproteus/Plasmodium* and HeamFL and HeamR2L for *Leucytozoon* [8-10].

Table S1. Coordinates of each sampling site. Coordinates are given in WGS84 projection.

Region	Region name	Plot number	X	Y
Southwest	Schwäbische Alb	4	9.245	48.399
Southwest	Schwäbische Alb	5	9.415	48.420
Southwest	Schwäbische Alb	6	9.446	48.394
Southwest	Schwäbische Alb	7	9.261	48.396
Southwest	Schwäbische Alb	8	9.382	48.383
Southwest	Schwäbische Alb	9	9.415	48.369
Southwest	Schwäbische Alb	18	9.234	48.365
Southwest	Schwäbische Alb	19	9.311	48.484
Southwest	Schwäbische Alb	20	9.319	48.370
Southwest	Schwäbische Alb	21	9.321	48.484
Southwest	Schwäbische Alb	22	9.453	48.382
Southwest	Schwäbische Alb	23	9.488	48.386
Southwest	Schwäbische Alb	24	9.295	48.479
Southwest	Schwäbische Alb	27	9.474	48.400
Southwest	Schwäbische Alb	41	9.404	48.364
Southwest	Schwäbische Alb	42	9.453	48.396
Southwest	Schwäbische Alb	43	9.378	48.379
Southwest	Schwäbische Alb	45	9.501	48.389
Southwest	Schwäbische Alb	48	9.473	48.388
Southwest	Schwäbische Alb	49	9.476	48.445
Southwest	Schwäbische Alb	50	9.254	48.392
Northeast	Schorfheide-Chorin	5	13.885	53.057
Northeast	Schorfheide-Chorin	6	13.842	52.907
Northeast	Schorfheide-Chorin	7	13.694	53.107
Northeast	Schorfheide-Chorin	8	13.930	53.192
Northeast	Schorfheide-Chorin	9	13.810	53.045
Northeast	Schorfheide-Chorin	35	13.853	52.911
Northeast	Schorfheide-Chorin	36	13.754	52.951
Northeast	Schorfheide-Chorin	37	13.783	52.940
Northeast	Schorfheide-Chorin	38	13.671	52.888
Northeast	Schorfheide-Chorin	39	13.866	52.925
Northeast	Schorfheide-Chorin	40	13.862	52.915
Northeast	Schorfheide-Chorin	41	13.910	52.912
Northeast	Schorfheide-Chorin	42	13.915	52.903
Northeast	Schorfheide-Chorin	43	13.928	52.901
Northeast	Schorfheide-Chorin	44	13.859	52.918
Northeast	Schorfheide-Chorin	45	13.838	53.045
Northeast	Schorfheide-Chorin	46	13.777	53.072
Northeast	Schorfheide-Chorin	47	13.771	53.071
Northeast	Schorfheide-Chorin	48	13.845	53.051
Northeast	Schorfheide-Chorin	49	13.891	52.885
Northeast	Schorfheide-Chorin	50	13.787	53.040

Table S2. Overview of predictor variables used in the analysis, the *grey shaded* area includes the predictor variables that are obtained by LiDAR.

Predictor variable	Abbreviation in models and in dataset	Explanation and source
Non LiDAR		
Silvicultural management index	SMI	Combined index consisting of two components; risk of stand loss (determined by tree species selection and stand age) and stand density (determined by removal and regeneration of the stand biomass). Source: internal data, for more information see Schall and Ammer [11].
Forest category	Habitat_category	Source: own, see materials and methods.
Distance to standing water (m)	water_standing_distance_m	Distance to nearest standing water body, in meters. Source: Digital Landscape Models for each region, which are the basis for topographical maps at a scale of 1:10,000.
Distance to flowing water (m)	water_flowing_distance_m	Distance to nearest flowing water, in meters. Source: Digital Landscape Models for each region, which are the basis for topographical maps at a scale of 1:10,000.
Size of standing water (m2)	water_standing_size_m2	Size of nearest standing water body, in square meters. Source: Digital Landscape Models for each region, which are the basis for topographical maps at a scale of 1:10,000.
Depth of leaf litter layer (cm)	leaflitter_depth_cm	Source: own, see materials and methods.
Average temperature	temperature_avg_14d	Average of daily temperatures up to 14 days before capture. All experimental plots are outfitted with measurement stations for climatic conditions. Temperature is measured 2 meters above the ground. Source: internal data.
Average relative humidity	relhum_avg_14d	Average of daily relative humidity up to 14 days before capture. All experimental plots are outfitted with measurement stations for climatic conditions. Relative humidity is measured 2 meters above the ground. Source: internal data.
Region	Explo	Region, either Northwest ('SEW') or Southeast ('AEW'), see materials and methods.
Year	Year	Year of capture, either 2014 or 2015, see materials and methods.
LiDAR		
Understory	An2	Proportion of understory vegetation, up to two meter above the ground. Source: internal data
Shrub layer	Sh	Proportion of shrub vegetation, between three and seven meter above the ground. Source: internal data
South facing	So	Proportion of south facing canopy. Source: internal data
Gap	Ga.1	Proportion of as gap; measured as where the outer canopy surface height is lower than the median outer canopy surface height within a local neighborhood minus the corresponding interquartile distance or less one meter. Source: internal data
Edge	Ed	Proportion of where the outer canopy surface differs 5m in height at 1m horizontal distance. Source: internal data
Open stem zone	Bo	Trunk space void of detected canopy material. Source: internal data
Entropy	En	Mean of entropy; the local vertical variation that is high for equal distributed echoes, low for clustered echoes; it is a parameter of texture. Source: internal data

Table S3. Overview of captured vector species grouped per family for the years 2014 and 2015. (O) ornithophilic; (M) mammalophilic; (O/M) either; (BP) study where blood meal resulted in detecting of Haemosporidia; (Mb) study where blood meal contained mammalian blood; (Ab) study where blood meal contained avian blood.

Family	Species	Affinity (reference)
Ceratopogonidae (Biting midges)	*Culicoides circumscriptus*	(O) [12-14], (O/M) [15], (BP) [16,17]
	Culicoides clastrieri	(Mb) [18], (Ab) [19],
	Culicoides duddingstoni	(O) [13,15]
	Culicoides festivipennis	(O) [12-15,17], (BP) [14,16,20], (Ab) [18]
	Culicoides impunctatus	(O) [21], (BP) [21]
	Culicoides kibunensis	(O/M) [15], (BP) [16,20], (Ab) [15,18,19]
	Culicoides lupicaris	(Mb) [13,15]
	Culicoides newsteadi	(Mb) [13,15]
	Culicoides obsoletus	(O/M) [22], (O) [17,18], (Ab) [22]
	Culicoides pictipennis	(O) [13,15,17,23], (BP)[16,19], (Ab) [18,19]
	Culicoides poperinghensis	(M) [15,18], (BP) [18]
	Culicoides pulicaris	(M) [13,15,22,24], (Ab) [25]
	Culicoides punctatus	(O) [17], (O/M) [22], (Mb) [13], (Ab) [13,22]
	Culicoides scoticus	(Mb) [13,18,22], (Ab) [22]
	Culicoides segnis	(BP) [16,20]
	Culicoides truncorum	(O) [23], (BP) [16]
Culicidae (mosquitoes)	*Aedes cantans*	(M) [26], (Mb) [27], (Ab) [28,29]
	Aedes cataphylla	(M) [30]
	Aedes cinereus	(O/M) [31], (Mb) [32], (Ab) [27,32]
	Aedes communis	(O/M) [31], (Mb) [27,32]
	Aedes diantaeus	(M) [33]
	Aedes hexodontus	Vector of viruses in mammals [34]
	Aedes leucomelas	NA
	Aedes punctor	Vector of viruses in mammals [34]
	Aedes riparius	(M) [26]
	Anopheles claviger	(Mb) [27,35,36], (Ab) [36]
	Anopheles plumbeus	(O) [37], (Mb) [27]
	Coquillettidia richiardii	(O) [26], (O/M) [36], (Mb) [27]
Simuliidae (black flies)	*Simuliidae (general)*	Vectors of Haemosporidia of the genus *Leucocytozoon* [2]
	Simulium intermedium	(Mb) [38]
	Simulium lundstromi	NA
	Simulium noelleri	(Mb) [38]
	Simulium vernum	(BP) [39], (Ab) [39]

Table S4. Number of female individuals caught per species in each region, separated per year.

	Species	*Southwest*		*Northeast*	
		2014	*2015*	*2014*	*2015*
Ceratopogonidae (Biting midges)	*Culicoides circumscriptus*				1
	Culicoides clastrieri			24	40
	Culicoides duddingstoni				3
	Culicoides festivipennis			163	993
	Culicoides impunctatus			77	1228
	Culicoides kibunensis				83
	Culicoides lupicaris	1		27	
	Culicoides newsteadi			15	107
	Culicoides obsoletus	3	2	7	
	Culicoides pictipennis			129	283
	Culicoides poperinghensis				17
	Culicoides pulicaris	3	1		
	Culicoides punctatus			19	247
	Culicoides scoticus	7		16	1
	Culicoides segnis			9	
	Culicoides truncorum				48
Culicidae (Mosquitoes)	*Aedes annulipes*			1	248
	Aedes cantans				541
	Aedes cataphylla			1	
	Aedes cinereus			3	24
	Aedes communis			2	59
	Aedes diantaeus				121
	Aedes leucomelas			1	
	Aedes punctor			2	61
	Aedes riparius			1	
	Anopheles claviger			1	6
	Anopheles plumbeus	14	2		15
	Coquillettidia richiardii			1	2
Simuliidae (black flies)	*Simulium intermedium*		1		
	Simulium lundstromi				4
	Simulium noelleri				1
	Simulium vernum	13	80		

Table S5. Overview of vector species with positive detection of haemosporidian infection after PCR.

Vector species	Region	Year	*Plasmodium or Haemoproteus*	*Leucocytozoon*	Forest Category
Anopheles plumbeus	SW	2014		X	Unmanaged
Anopheles plumbeus	SW	2014		X	Age class - young
Culicoides lupicaris	SW	2014	X		Age class - young
Simulium vernum	SW	2014		X	Unmanaged
Simulium vernum	SW	2014	X	X	Unmanaged
Simulium vernum	SW	2014		X	Age class - young
Simulium intermedium	SW	2015		X	Age class - old
Simulium vernum	SW	2015	X	X	Unmanaged
Simulium vernum	SW	2015		X	Age class - old
Simulium vernum	SW	2015	X		Unmanaged
Aedes cinereus	NE	2014		X	Unmanaged
Culicoides festivipennis	NE	2014	X		Age class - old
Culicoides festivipennis	NE	2014		X	Age class - young
Culicoides pictipennis	NE	2014	X		Unmanaged
Culicoides pictipennis	NE	2014	X		Age class - young
Culicoides pictipennis	NE	2014	X		Age class - old
Culicoides punctatus	NE	2014	X		Age class - old
Aedes annulipes	NE	2015	X		Age class - old
Aedes annulipes	NE	2015	X		Age class - young
Aedes annulipes	NE	2015	X		Age class - young
Aedes annulipes	NE	2015	X		Age class - young
Aedes annulipes	NE	2015	X		Age class - old
Aedes annulipes	NE	2015	X		Unmanaged
Aedes cantans	NE	2015	X		Age class - young
Aedes cantans	NE	2015	X		Age class - old
Aedes cantans	NE	2015	X		Age class - young
Aedes cantans	NE	2015	X		Age class - young
Aedes cantans	NE	2015	X		Age class - old
Aedes cantans	NE	2015	X		Unmanaged
Aedes cantans	NE	2015	X		Unmanaged
Aedes cinereus	NE	2015	X		Age class - young
Aedes cinereus	NE	2015	X		Unmanaged
Aedes cinereus	NE	2015	X		Unmanaged
Aedes cinereus	NE	2015	X		Age class - old
Aedes communis	NE	2015	X		Age class - young
Aedes communis	NE	2015	X		Age class - old
Aedes diantaeus	NE	2015	X		Age class - old
Aedes punctor	NE	2015	X		Age class - young
Aedes punctor	NE	2015	X		Unmanaged

Aedes punctor	NE	2015	X		Unmanaged
Aedes punctor	NE	2015	X		Unmanaged
Aedes punctor	NE	2015	X		Age class - young
Anopheles claviger	NE	2015	X		Age class - old
Anopheles claviger	NE	2015	X		Age class - young
Anopheles claviger	NE	2015	X		Unmanaged
Anopheles plumbeus	NE	2015	X		Age class - young
Anopheles plumbeus	NE	2015	X		Age class - old
Anopheles plumbeus	NE	2015	X		Unmanaged
Anopheles plumbeus	NE	2015	X		Unmanaged
Anopheles plumbeus	NE	2015	X		Age class - old
Coquillettidia richiardii	NE	2015	X		Age class - old
Culicoides circumscriptus	NE	2015	X		Age class - young
Culicoides festivipennis	NE	2015	X		Age class - young
Culicoides festivipennis	NE	2015	X		Age class - young
Culicoides festivipennis	NE	2015	X	X	Age class - young
Culicoides festivipennis	NE	2015	X		Age class - old
Culicoides festivipennis	NE	2015	X		Unmanaged
Culicoides festivipennis	NE	2015	X		Unmanaged
Culicoides festivipennis	NE	2015	X		Unmanaged
Culicoides festivipennis	NE	2015	X		Age class - young
Culicoides impunctatus	NE	2015	X		Age class - young
Culicoides impunctatus	NE	2015	X		Age class - old
Culicoides impunctatus	NE	2015	X		Age class - young
Culicoides impunctatus	NE	2015	X		Unmanaged
Culicoides impunctatus	NE	2015	X		Unmanaged
Culicoides impunctatus	NE	2015	X		Age class - young
Culicoides kibunensis	NE	2015	X		Age class - old
Culicoides newsteadi	NE	2015	X		Unmanaged
Culicoides newsteadi	NE	2015	X		Age class - old
Culicoides pictipennis	NE	2015	X		Age class - old
Culicoides pictipennis	NE	2015	X		Age class - old
Culicoides pictipennis	NE	2015	X		Age class - young
Culicoides pictipennis	NE	2015	X		Age class - old
Culicoides pictipennis	NE	2015	X		Unmanaged
Culicoides pictipennis	NE	2015	X		Unmanaged
Culicoides pictipennis	NE	2015	X		Unmanaged
Culicoides pictipennis	NE	2015	X		Unmanaged
Culicoides pictipennis	NE	2015	X		Age class - young
Culicoides pictipennis	NE	2015	X		Age class - old
Culicoides poperinghensis	NE	2015	X		Age class - old
Culicoides punctatus	NE	2015	X		Unmanaged
Culicoides truncorum	NE	2015	X		Unmanaged
Simulium lundstromi	NE	2015	X		Age class - young
Simulium noelleri	NE	2015	X		Age class - young

Table S6. Model estimates from the Generalized Linear Models for total vector abundance. The best-fitted model was selected based on lowest AICc and is indicated by grey fields. All parameters were fitted as fixed factors. Shown are parameter estimates of the separate models (*blank fields* indicate parameter was excluded in further model selection; significant parameters are highlighted in ***bold***, GLM $P < 0.05$). Inclusion of categorical variables are indicated by a plus (+) sign. Explanation of variables abbreviations are in Table S1.

	Variable name																		*Statistical parameters*			
**Model name**	**SMI**	**Forest category**	**Understory**	**Shrub layer**	**South facing**	**Gap**	**Edge**	**Open stem zone**	**Entropy**	**water standing distance (km)**	**water flowing distance (km)**	**water standing size (ha)**	**leaf litter depth (cm)**	**Temperature (average over 14 d)**	**Relative humidity (average over 14d)**	**Genus**	**Year**	**Region**	**Degrees of freedom**	**AICc**	**ΔAICc**	**Weight**
ab.9		***(+)***			***-21.68***	***17.18***		***-0.17***	***-1.03***					***-0.21***	***-0.05***	***(+)***	***0.68***	***(+)***	16.00	1192.77	0.00	0.46
ab.8		***(+)***			***-21.63***	***17.61***		***-0.18***	***-1.11***			0.01		***-0.21***	***-0.05***	***(+)***	***0.63***	***(+)***	17.00	1193.94	1.18	0.25
ab.7		***(+)***			***-19.79***	***14.88***		***-0.17***	***-1.22***		0.27	0.01		***-0.24***	***-0.04***	***(+)***	***0.70***	***(+)***	18.00	1194.53	1.77	0.19
ab.6	2.18	***(+)***			***-20.40***	***14.44***		***-0.15***	-1.01		0.33	0.01		***-0.24***	***-0.04***	***(+)***	***0.66***	***(+)***	19.00	1196.49	3.72	0.07
ab.5	2.48	***(+)***			***-21.02***	***14.61***		***-0.15***	-0.96		0.35	0.01	-0.07	***-0.24***	***-0.04***	***(+)***	0.55	***(+)***	20.00	1198.95	6.19	0.02
ab.4	2.35	***(+)***	0.49		***-20.48***	***14.51***		***-0.15***	-1.04		0.35	0.01	-0.07	***-0.24***	***-0.04***	***(+)***	0.57	***(+)***	21.00	1201.52	8.76	0.01
ab.3	2.28	***(+)***	0.52		***-20.23***	***14.43***		***-0.15***	-1.03	-0.04	0.35	0.01	-0.08	***-0.24***	***-0.04***	***(+)***	0.56	***(+)***	22.00	1204.18	11.41	0.00
ab.2	2.18	***(+)***	0.66	0.39	***-19.91***	***14.37***		-0.14	-1.05	-0.04	0.36	0.01	-0.07	***-0.24***	***-0.04***	***(+)***	0.59	***(+)***	23.00	1206.88	14.11	0.00
ab.1	2.17	***(+)***	0.66	0.43	***-19.84***	***14.49***	-0.13	-0.14	-1.07	-0.04	0.36	0.01	-0.07	***-0.24***	***-0.04***	***(+)***	0.59	(+)	24.00	1209.63	16.87	0.00

Table S7. Model estimates from the Generalized Linear Models for female ceratopogonid vector abundance. The best-fitted model was selected based on lowest AICc and is indicated by grey fields. All parameters were fitted as fixed factors. Shown are parameter estimates of the separate models (*blank fields* indicate parameter was excluded in further model selection; significant parameters are highlighted in **bold**, GLM $P < 0.05$). Inclusion of categorical variables are indicated by a plus (+) sign. Explanation of variables abbreviations are in Table S1.

	Variable name																		*Statistical parameters*			
**Model name**	**SMI**	**Forest category**	**Understory**	**Shrub layer**	**South facing**	**Gap**	**Edge**	**Open stem zone**	**Entropy**	**water standing distance (km)**	**water flowing distance (km)**	**water standing size (ha)**	**leaf litter depth (cm)**	**Temperature (average over 14 d)**	**Relative humidity (average over 14d)**	**Genus**	**Year**	**Region**	**Degrees of freedom**	**AICc**	**ΔAICc**	**Weight**
cera.11				***8.59***		***25.63***	***-26.25***		***-4.86***					***-0.26***	***-0.08***			***(+)***	9.00	728.57	0.00	0.44
cera.10				***10.02***	-8.27	***30.43***	***-26.06***		***-5.42***					***-0.28***	***-0.08***			***(+)***	10.00	729.50	0.92	0.28
cera.9				***9.91***	-10.86	***27.90***	***-25.80***		***-5.60***		0.25			***-0.32***	***-0.08***			***(+)***	11.00	730.33	1.76	0.18
cera.8		(+)		***10.72***	-9.87	***29.10***	***-25.25***		***-5.73***		0.35			***-0.31***	***-0.08***			***(+)***	13.00	733.86	5.28	0.03
cera.7	5.19	***(+)***		***7.96***	***-13.58***	***24.63***	***-20.46***		***-4.39***		0.39			***-0.26***	***-0.07***			***(+)***	14.00	734.19	5.62	0.03
cera.6	5.61	***(+)***		***10.34***	-11.19	***28.11***	***-26.36***		***-5.82***		***0.58***	0.02		***-0.28***	***-0.08***			***(+)***	15.00	734.97	6.40	0.02
cera.5	5.82	***(+)***		***12.08***	-10.19	***31.90***	***-30.54***		***-6.83***	0.49	***0.58***	***0.02***		***-0.24***	***-0.07***			***(+)***	16.00	736.09	7.52	0.01
cera.4	5.87	***(+)***		***13.93***	-11.69	***38.17***	***-33.20***		***-7.60***	***0.69***	0.44	***0.02***	0.25	***-0.19***	***-0.08***			***(+)***	17.00	736.56	7.99	0.01
cera.3	4.85	***(+)***		***14.87***	-12.24	***39.66***	***-34.00***		***-8.04***	***0.68***	0.43	***0.03***	0.40	***-0.20***	***-0.08***		0.41	***(+)***	18.00	739.21	10.63	0.00
cera.2	3.74	***(+)***	1.45	***15.32***	-11.42	***39.67***	***-34.42***		***-8.31***	0.58	0.46	***0.03***	0.37	***-0.23***	***-0.07***		0.58	***(+)***	19.00	741.90	13.32	0.00
cera.1	3.48	***(+)***	1.63	***18.29***	-7.47	***40.38***	***-37.15***	0.07	***-9.01***	0.64	0.50	***0.03***	0.47	***-0.22***	***-0.07***		0.76	***(+)***	20.00	744.78	16.21	0.00

Table S8. Model estimates from the Generalized Linear Models for female culicid vector abundance. The best-fitted model was selected based on lowest AICc and is indicated by grey fields. All parameters were fitted as fixed factors. Shown are parameter estimates of the separate models (*blank fields* indicate parameter was excluded in further model selection; significant parameters are highlighted in **bold**, GLM *P* < 0.05). Inclusion of categorical variables are indicated by a plus (+) sign. Explanation of variables abbreviations are in Table S1.

Variable name																			*Statistical parameters*			
**Model name**	**SMI**	**Forest category**	**Understory**	**Shrub layer**	**South facing**	**Gap**	**Edge**	**Open stem zone**	**Entropy**	**water standing distance (km)**	**water flowing distance (km)**	**water standing size (ha)**	**leaf litter depth (cm)**	**Temperature (average over 14 d)**	**Relative humidity (average over 14d)**	**Genus**	**Year**	**Region**	**Degrees of freedom**	**AICc**	**ΔAICc**	**Weight**
culi.12	***-11.40***	***(+)***					3.62					***0.03***	***-0.63***	-0.15					9.00	341.31	0.00	0.69
culi.11	***-12.76***	***(+)***					3.60	-0.04				***0.03***	***-0.61***	-0.15					10.00	343.59	2.28	0.22
culi.10	***-13.38***	***(+)***			-15.58		10.30	-0.08				0.02	***-0.66***	-0.17					11.00	345.78	4.47	0.07
culi.9	***-13.61***	***(+)***			-18.18		11.72	-0.09				0.02	***-0.56***	-0.15			0.38		12.00	348.79	7.48	0.02
culi.8	***-13.42***	(+)			-26.72	6.59	12.34	-0.14				0.02	***-0.56***	-0.17			0.46		13.00	351.96	10.65	0.00
culi.7	***-13.58***	(+)			-29.17	7.24	13.39	-0.15		-0.08		0.02	***-0.54***	-0.16			0.49		14.00	355.48	14.17	0.00
culi.6	***-12.88***	(+)			-32.57	8.79	14.09	-0.16		-0.12		0.02	***-0.55***	-0.16	-0.01		0.41		15.00	359.16	17.85	0.00
culi.5	***-12.80***	(+)		2.08	-33.11	12.54	12.24	-0.13		-0.19		0.02	***-0.54***	-0.20	-0.01		0.38		16.00	362.93	21.62	0.00
culi.4	***-12.57***	(+)	-1.39	2.32	-35.93	16.14	10.92	-0.15		-0.29		0.01	***-0.54***	-0.26	-0.02		0.39		17.00	366.88	25.56	0.00
culi.3	***-13.24***	(+)	-1.41	2.17	-34.11	15.73	10.29	-0.16		-0.24	-0.15	0.01	***-0.54***	-0.24	-0.02		0.39		18.00	371.15	29.84	0.00
culi.2	***-12.86***	(+)	-1.89	1.93	-36.29	16.84	10.11	-0.17		-0.16	-0.13	0.01	***-0.54***	-0.24	-0.02		0.36	(+)	19.00	375.72	34.41	0.00
culi.1	-11.32	(+)	-2.62	1.92	-40.51	17.57	11.02	-0.18	0.39	-0.18	-0.11	0.01	***-0.54***	-0.26	-0.03		0.37	(+)	20.00	380.57	39.25	0.00
culi.15		***(+)***										***0.03***	***-0.82***						6.00	420.34	79.02	0.00
culi.14	-5.48	***(+)***										***0.03***	***-0.77***						7.00	421.36	80.05	0.00
culi.13	-7.23	***(+)***					2.28					***0.03***	***-0.72***						8.00	423.08	81.77	0.00

Table S9. Model estimates from the Generalized Linear Models for prevalence inside the vector. The best-fitted model was selected based on lowest AICc and is indicated by grey fields. All parameters were fitted as fixed factors. Shown are parameter estimates of the separate models (*blank fields* indicate parameter was excluded in further model selection; significant parameters are highlighted in **bold**, GLM *P* < 0.05). Inclusion of categorical variables are indicated by a plus (+) sign. Explanation of variables abbreviations are in Table S1.

	Variable name																		Statistical parameters			
**Model name**	**SMI**	**Forest category**	**Understory**	**Shrub layer**	**South facing**	**Gap**	**Edge**	**Open stem zone**	**Entropy**	**water standing distance (km)**	**water flowing distance (km)**	**water standing size (ha)**	**leaf litter depth (cm)**	**Temperature (average over 14 d)**	**Relative humidity (average over 14d)**	**Genus**	**Year**	**Region**	**Degrees of freedom**	**AICc**	**ΔAICc**	**Weight**
pr.11	***20.49***	***(+)***		***-5.79***			***14.38***		***5.93***				***-0.42***		***-0.05***	***(+)***			13.00	198.05	0.00	0.33
pr.10	***22.50***	***(+)***	-2.61	***-7.47***			***16.05***		***7.15***				***-0.40***		***-0.05***	***(+)***			14.00	198.72	0.67	0.24
pr.9	***20.76***	***(+)***	-3.22	***-12.50***			***16.88***	-0.15	***7.66***				***-0.41***		-0.04	(+)			15.00	199.48	1.43	0.16
pr.8	***23.96***	***(+)***	-3.82	***-16.66***	-16.77		***23.77***	-0.29	***8.51***				***-0.43***		-0.05	(+)			16.00	200.36	2.31	0.10
pr.7	***21.42***	***(+)***	-4.10	***-17.60***	-14.78		***23.53***	-0.32	***8.44***		-0.48		-0.38		***-0.05***	***(+)***			17.00	201.43	3.37	0.06
pr.4	***23.03***	***(+)***	***-6.29***	***-19.09***	-28.13	12.66	***22.68***	***-0.45***	***9.04***		-0.74	-0.01	-0.37	-0.10	***-0.05***	(+)			20.00	201.84	3.79	0.05
pr.6	***22.08***	***(+)***	***-5.15***	***-18.92***	-20.51		***27.93***	***-0.35***	***9.45***		-0.59	-0.02	-0.36		***-0.05***	***(+)***			18.00	202.76	4.71	0.03
pr.3	***22.27***	***(+)***	***-6.55***	***-18.48***	-27.16	12.71	***22.20***	***-0.44***	***8.97***	-0.14	-0.69	-0.01	-0.37	-0.11	***-0.05***	(+)			21.00	204.30	6.24	0.01
pr.5	***22.08***	***(+)***	***-4.85***	***-17.96***	-22.99	7.60	***25.46***	***-0.38***	***9.03***		-0.66	-0.01	-0.36		***-0.05***	***(+)***			19.00	205.03	6.97	0.01
pr.2	***22.10***	***(+)***	***-6.08***	***-19.66***	-27.08	9.99	***25.41***	***-0.44***	***9.63***	-0.27	-0.72	-0.02	-0.40	-0.12	***-0.05***	(+)		(+)	22.00	206.77	8.72	0.00
pr.1	22.30	(+)	-6.13	-19.82	-27.32	9.94	25.57	-0.44	9.71	-0.27	-0.72	-0.02	-0.41	-0.12	-0.05	(+)	-0.05	(+)	23.00	209.49	11.43	0.00

References cited in Supplementary Information

1 Atkinson, C. T. in *Field manual of wildlife diseases: general field procedures and diseases of birds. Biological Resources Division Information and Technology Report, 2001* 193-199 (1999).

2 Valkiūnas, G. *Avian malaria parasites and other haemosporidia*. (CRC Press, 2005).

3 Hubálek, Z. An annotated checklist of pathogenic microorganisms associated with migratory birds. *Journal of Wildlife Diseases* **40**, 639-659 (2004).

4 Martinsen, E. S., Perkins, S. L. & Schall, J. J. A three-genome phylogeny of malaria parasites (Plasmodium and closely related genera): evolution of life-history traits and host switches. *Molecular phylogenetics and evolution* **47**, 261-273, doi:10.1016/j.ympev.2007.11.012 (2008).

5 Senar, J. C., Copetel, J. L., Domenechi, J. & Walter, G. V. O. N. Prevalence of louse-flies, Diptera, Hippoboscidae, parasiting a cardueline finch and its effect on body condition. *Ardea* **82**, 157-160 (1994).

6 Folmer, O., Black, M., Hoeh, W., Lutz, R. & Vrijenhoek, R. DNA primers for amplification of mitochondrial cytochrome c oxidase subunit I from diverse metazoan invertebrates. *Molecular Marine Biology and Biotechnology* **3**, 294-299, doi:10.1371/journal.pone.0013102 (1994).

7 Martínez-de la Puente, J. *et al.* Genetic characterization and molecular identification of the bloodmeal sources of the potential bluetongue vector Culicoides obsoletus in the Canary Islands, Spain. *Parasites & vectors* **5**, 147-147, doi:10.1186/1756-3305-5-147 (2012).

8 Hellgren, O., Waldenström, J. & Bensch, S. A new PCR assay for simultaneous studies of Leucocytozoon, Plasmodium, and Haemoproteus from avian blood. *Journal of Parasitology* **90**, 797-802, doi:10.1645/GE-184R1 (2004).

9 Bensch, S. *et al.* Host specificity in avian blood parasites: a study of Plasmodium and Haemoproteus mitochondrial DNA amplified from birds. *Proceedings. Biological sciences / The Royal Society* **267**, 1583-1589, doi:10.1098/rspb.2000.1181 (2000).

10 Waldenström, J., Bensch, S., Hasselquist, D. & Östman, Ö. A new nested polymerase chain reaction method very efficient in detecting *Plasmodium* and *Haemoproteus* infections from avian blood. *Journal of Parasitology* **90**, 191-194 (2004).

11 Schall, P. & Ammer, C. How to quantify forest management intensity in Central European forests. *European Journal of Forest Research* **132**, 379-396, doi:10.1007/s10342-013-0681-6 (2013).

12 Foxi, C. & Delrio, G. Larval habitats and seasonal abundance of Culicoides biting midges found in association with sheep in northern Sardinia, Italy. *Medical and veterinary entomology* **24**, 199-209, doi:10.1111/j.1365-2915.2010.00861.x (2010).

13 Pettersson, E. *et al.* Molecular identification of bloodmeals and species composition in Culicoides biting midges. *Medical and veterinary entomology* **27**, 104-112, doi:10.1111/j.1365-2915.2012.01038.x (2013).

14 Bobeva, A., Ilieva, M., Dimitrov, D. & Zehtindjiev, P. Degree of associations among vectors of the genus Culicoides (Diptera: Ceratopogonidae) and host bird species with respect to haemosporidian parasites in NE Bulgaria. *Parasitology research* **113**, 4505-4511, doi:10.1007/s00436-014-4140-1 (2014).

15 Lassen, S. B., Nielsen, S. A. & Kristensen, M. Identity and diversity of blood meal hosts of biting midges (Diptera: Ceratopogonidae: Culicoides Latreille) in Denmark. *Parasites & vectors* **5**, 143-143, doi:10.1186/1756-3305-5-143 (2012).

16 Martínez-de la Puente, J., Martínez, J., Aguilar, J. R. d., Herrero, J. & Merino, S. On the specificity of avian blood parasites: revealing specific and generalist relationships between haemosporidians and biting midges. *Molecular ecology* (2011).

17 Bobeva, A., Zehtindjiev, P., Bensch, S. & Radrova, J. A survey of biting midges of the genus Culicoides Latreille, 1809 (Diptera: Ceratopogonidae) in NE Bulgaria, with respect to transmission of avian haemosporidians. *Acta parasitologica / Witold Stefański Institute of Parasitology, Warszawa, Poland* **58**, 585-591, doi:10.2478/s11686-013-0185-z (2013).
18 Santiago-Alarcon, D., Havelka, P., Schaefer, H. M. & Segelbacher, G. Bloodmeal analysis reveals avian Plasmodium infections and broad host preferences of Culicoides (Diptera: Ceratopogonidae) vectors. *PloS one* **7**, e31098-e31098, doi:10.1371/journal.pone.0031098 (2012).
19 Santiago-Alarcon, D., Havelka, P., Pineda, E., Segelbacher, G. & Schaefer, H. M. Urban forests as hubs for novel zoonosis: blood meal analysis, seasonal variation in Culicoides (Diptera: Ceratopogonidae) vectors, and avian haemosporidians. *Parasitology*, 1-12, doi:10.1017/S0031182013001285 (2013).
20 Synek, P., Munclinger, P., Albrecht, T. & Votýpka, J. Avian haemosporidians in haematophagous insects in the Czech Republic. *Parasitology research* **112**, 839-845, doi:10.1007/s00436-012-3204-3 (2013).
21 Valkiūnas, G. & Iezhova, T. A. Detrimental Effects of Haemoproteus Infections on the Survival of Biting Midge Culicoides impunctatus (Ditpera: Ceratopogonidae). *The Journal of parasitology* **90**, 194-196 (2004).
22 Lassen, S. B., Nielsen, S. a., Skovgård, H. & Kristensen, M. Molecular identification of bloodmeals from biting midges (Diptera: Ceratopogonidae: Culicoides Latreille) in Denmark. *Parasitology research* **108**, 823-829, doi:10.1007/s00436-010-2123-4 (2011).
23 Votýpka, J., Synek, P. & Svobodová, M. Endophagy of biting midges attacking cavity-nesting birds. *Medical and Veterinary Entomology* **23**, 277-280, doi:10.1111/j.1365-2915.2009.00800.x (2009).
24 Ninio, C., Augot, D., Delecolle, J. C., Dufour, B. & Depaquit, J. Contribution to the knowledge of Culicoides (Diptera: Ceratopogonidae) host preferences in France. *Parasitology Research* **108**, 657-663, doi:10.1007/s00436-010-2110-9 (2011).
25 Calvo, J. H. *et al.* Host feeding patterns of Culicoides species (Diptera: Ceratopogonidae) within the Picos de Europa National Park in northern Spain. *Bulletin of Entomological Research*, 1-6, doi:10.1017/S0007485312000284 (2012).
26 Glaizot, O. *et al.* High prevalence and lineage diversity of avian malaria in wild populations of great tits (Parus major) and mosquitoes (Culex pipiens). *PLoS ONE* **7**, doi:10.1371/journal.pone.0034964 (2012).
27 Börstler, J. *et al.* Host-feeding patterns of mosquito species in Germany. *Parasites & Vectors* **9**, 318-318, doi:10.1186/s13071-016-1597-z (2016).
28 Bernotienė, R. The fauna and seasonal activity of mosquitoes (Diptera: Culicidae) in the Curonian Spit (Russia, Lithuania). *European Mosquito Bulletin* **30**, 72-78 (2012).
29 Valkiunas, G., Kazlauskiene, R., Bernotiene, R., Palinauskas, V. & Iezhova, T. A. Abortive long-lasting sporogony of two Haemoproteus species (Haemosporida, Haemoproteidae) in the mosquito Ochlerotatus cantans, with perspectives on haemosporidian vector research. *Parasitology Research* **112**, 2159-2169, doi:10.1007/s00436-013-3375-6 (2013).
30 Service, M. W. in *Medical Insects and Arachnids* 120-240 (Chapman & Hall, 1993).
31 Turell, M. J., Lundstrom, J. O. & Niklasson, B. Transmission of Ockelbo virus by Aedes cinereus, Ae. communis, and Ae. excrucians (Diptera: Culicidae) collected in an enzootic area in central Sweden. *J Med Entomol* **27**, 266-268 (1990).
32 Molaei, G., Andreadis, T. G., Armstrong, P. M. & Diuk-Wasser, M. Host-feeding patterns of potential mosquito vectors in Connecticut, U.S.A.: molecular analysis of bloodmeals from 23 species of Aedes, Anopheles, Culex, Coquillettidia, Psorophora, and Uranotaenia. *Journal of medical entomology* **45**, 1143-1151, doi:10.1603/0022-2585(2008)45 (2008).
33 Jaenson, T. G. Attraction to mammals of male mosquitoes with special reference to Aedes diantaeus in Sweden. *Journal of the American Mosquito Control Association* **1**, 195-198 (1985).

34 Hubálek, Z. Mosquito-borne viruses in Europe. *Parasitology Research* **103**, doi:10.1007/s00436-008-1064-7 (2008).

35 Becker, N. *et al. Mosquitoes and Their Control*. (Springer, 2010).

36 Ventim, R. *et al.* Avian malaria infections in western European mosquitoes. *Parasitology research* **111**, 637-645, doi:10.1007/s00436-012-2880-3 (2012).

37 Cerný, O., Votýpka, J. & Svobodová, M. Spatial feeding preferences of ornithophilic mosquitoes, blackflies and biting midges. *Medical and veterinary entomology* **25**, 104-108, doi:10.1111/j.1365-2915.2010.00875.x (2011).

38 Malmqvist, B., Strasevicius, D., Hellgren, O., Adler, P. H. & Bensch, S. Vertebrate host specificity of wild-caught blackflies revealed by mitochondrial DNA in blood. *Proceedings. Biological sciences / The Royal Society* **271 Suppl**, S152-155, doi:10.1098/rsbl.2003.0120 (2004).

39 Synek, P. *et al.* Haemosporidian infections in the Tengmalm's Owl (Aegolius funereus) and potential insect vectors of their transmission. *Parasitology Research* **115**, 291-298, doi:10.1007/s00436-015-4745-z (2016).

Supplementary Information Figures

Figure S1. The effect of the significant predictor variables on the abundance of ceratopogonid vectors (±95% CI). Shown are values predicted using the best-fitted model from Table 1.

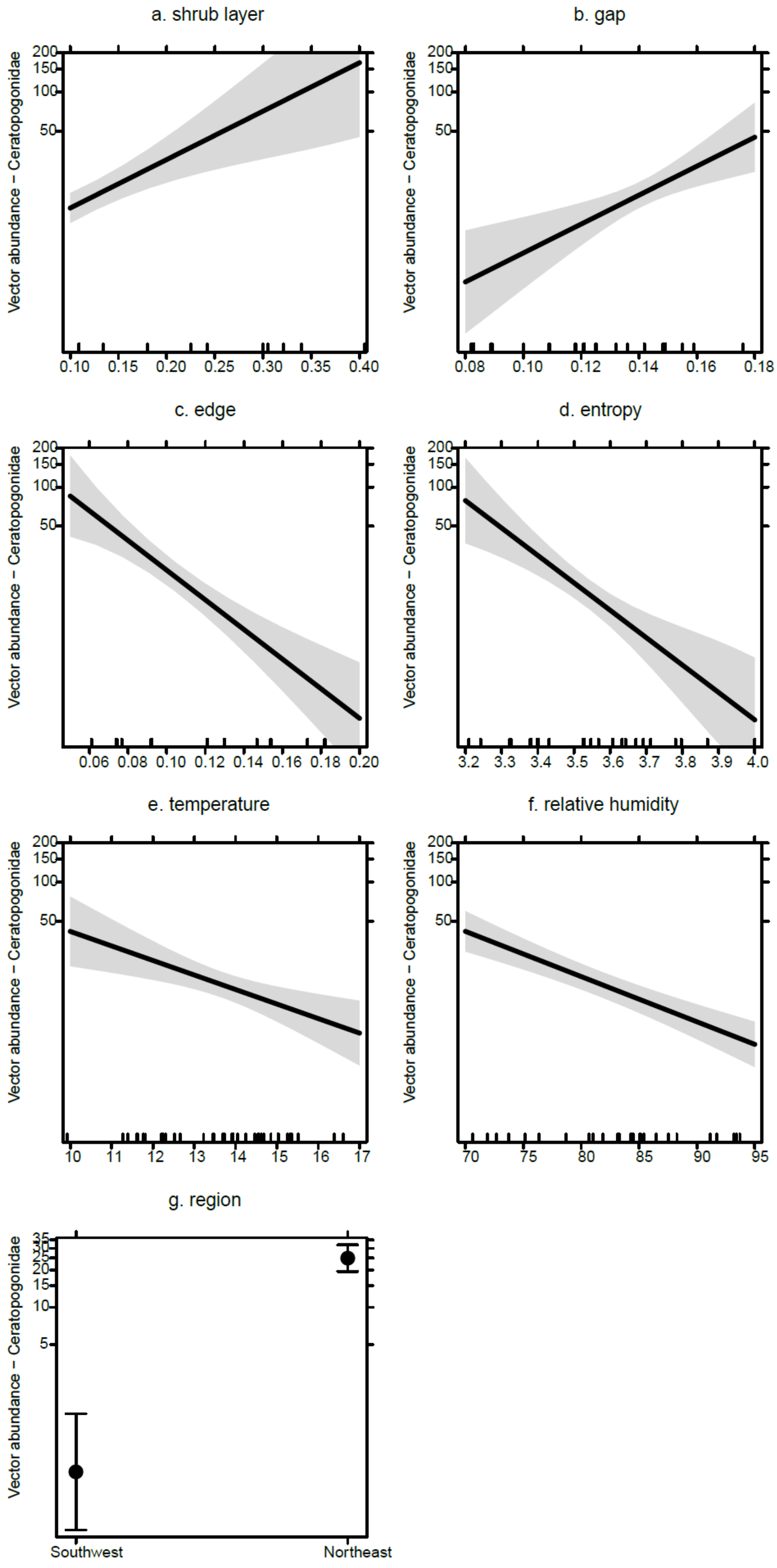

Figure S2. The effect of the significant predictor variables on the abundance of culicid vectors (±95% CI). Shown are values predicted using the best-fitted model from Table 1.

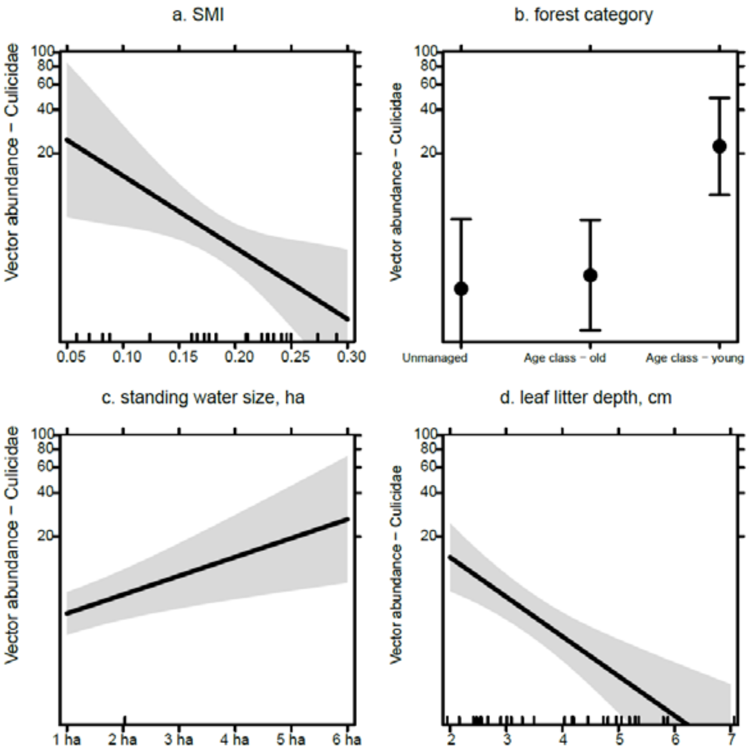

Fig. S3. Relationships between the Silvicultural Management Index (SMI), forest categories and leaf litter depth for the sampled forest plots. *(a):* Boxplot between the Silvicultural Management Index (SMI) and the forest category. The unmanaged category contains forest plots with a significant lower SMI than the age class (managed) forest classes (Kruskal Wallis, $\chi 2=74.036$, df=37, $P < 0.01$). *(b):* Boxplot between the leaf litter depth and the forest category. There are no significant differences between the leaf litter depths and the forest categories (Kruskal Wallis, $\chi 2=75$, df=73, $P = 0.413$). *Fig. (c):* Scatterplot between the leaf litter depth and silvicultural management index (SMI). There is no significant relationship between the leaf litter depth and the SMI (GLM, $P = 0.343$).

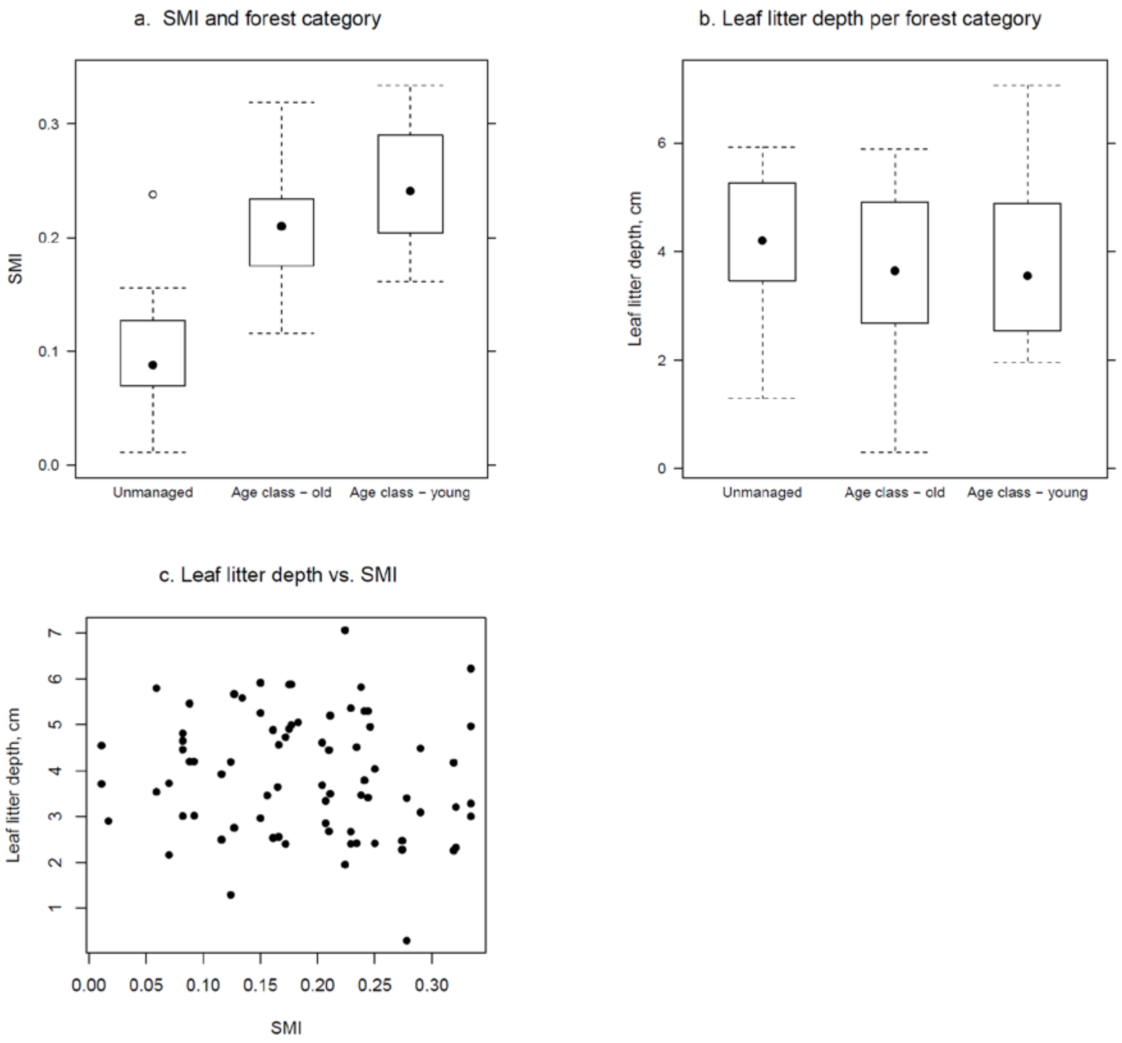

Supplement: Supplementary file 1 — Supplementary Information [file 41598_2019_45068_MOESM1_ESM.pdf]
